# Influence of Land Cover and Soil Moisture based Brown Ocean Effect on an Extreme Rainfall Event from a Louisiana Gulf Coast Tropical System

**DOI:** 10.1038/s41598-019-53031-6

**Published:** 2019-11-20

**Authors:** Udaysankar S. Nair, Eric Rappin, Emily Foshee, Warren Smith, Roger A. Pielke, Rezaul Mahmood, Jonathan L. Case, Clay B. Blankenship, Marshall Shepherd, Joseph A. Santanello, Dev Niyogi

**Affiliations:** 1 Department of Atmospheric Science, University of Alabama in Huntsville, Huntsville, AL 35806 USA; 2 Department of Geography and Geology and Kentucky Climate Center, Western Kentucky University, Bowling Green, KY 42101 USA; 3 Atmospheric Chemistry Observations and Modeling Laboratory, National Center for Atmospheric Research, Boulder, CO 80301 USA; 4 Cooperative Institute for Research in Environmental Sciences, University of Colorado Boulder, Boulder, CO 80309 USA; 5 High Plains Regional Climate Center, School of Natural Resources, University of Nebraska-Lincoln, Lincoln, NE 68583 USA; 6 ENSCO, Inc./NASA Short-term Prediction Research and Transition (SPoRT) Center, Huntsville, USA; 7 Universities Space Research Association, NASA Short-term Prediction Research and Transition (SPoRT) Center, Huntsville, USA; 8 University of Georgia, Department of Geography, Atmospheric Sciences Program, Athens, GA, USA; 9 NASA-GSFC, Hydrological Sciences Laboratory, Greenbelt, MD USA; 10 Department of Agronomy and Department of Earth, Atmospheric and Planetary Sciences, Purdue University, West Lafayette, IN 47907 USA

**Keywords:** Atmospheric dynamics, Natural hazards

## Abstract

Extreme flooding over southern Louisiana in mid-August of 2016 resulted from an unusual tropical low that formed and intensified over land. We used numerical experiments to highlight the role of the ‘Brown Ocean’ effect (where saturated soils function similar to a warm ocean surface) on intensification and it’s modulation by land cover change. A numerical modeling experiment that successfully captured the flood event (control) was modified to alter moisture availability by converting wetlands to open water, wet croplands, and dry croplands. Storm evolution in the control experiment with wet antecedent soils most resembles tropical lows that form and intensify over oceans. Irrespective of soil moisture conditions, conversion of wetlands to croplands reduced storm intensity, and also, non-saturated soils reduced rain by 20% and caused shorter durations of high intensity wind conditions. Developing agricultural croplands and more so restoring wetlands and not converting them into open water can impede intensification of tropical systems that affect the area.

## Introduction

**Figure 1:**
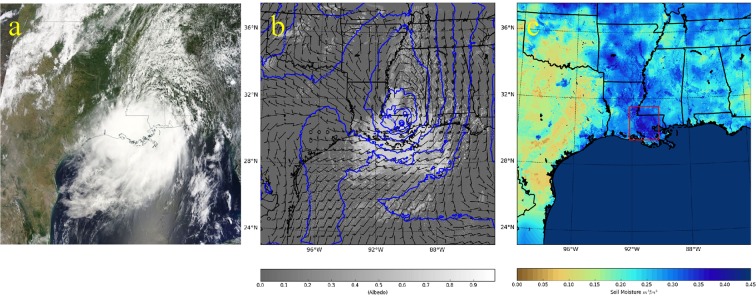
(**a**) True color composite of the tropical disturbance generated using data acquired by Moderate Resolution Imaging Spectroradiometer (MODIS) on the NASA Terra satellite platform^35^ (~1630 UTC LST) on 12 August 2016; (**b**) Albedo computed using the 1600 UTC instantaneous shortwave radiation fields in the 3 km spacing inner grid in the control experiment. Model-simulated cloud fields appear as bright features. Overlaid on the albedo fields are model-simulated geopotential height fields (blue) and 850 hPa wind barbs, also valid 12 August 2016; (**c**) NASA SPoRT soil moisture product used to initialize WRF. The red rectangle marks the region of high antecedent soil moisture conditions that potentially modulated the development of the tropical system. Rainfall averaged over this 2° × 2° rectangular region is used to intercompare the different numerical modeling experiments. Maps were created using Matplotlib, version 1.5.3^36^.

A tropical disturbance formed over the southern part of Louisiana in mid-August 2016 which interacted with an eastward-moving upper-level baroclinic trough, leading to the intensification of the system and a major flood disaster^1^. This system appeared to have all of the characteristics of a tropical depression, as observed in satellite imagery and in the wind field (Fig. 1a,b). Weak steering level winds, coupled with moisture, high convective available potential energy (CAPE) and a low convective inhibition (CIN) environment led to a relatively stationary system which caused local, intense rainfall over the region for several hours. Storm total accumulations from this system exceeded 780 mm (~5 times the long-term average rainfalls of 148 mm for Baton Rouge for the entire month of August) in southern Louisiana, and early estimates suggest economic losses of about $8.7 billion^2^. Typically, storms such as these are remnants of decaying tropical systems that form over the ocean and propagate onshore. In this respect, this event was unusual since the tropical depression developed and persisted over land.

Conceptually, tropical cyclones (including depressions) can be viewed as heat engines powered by surface enthalpy fluxes^3^. For the heat engine to function, heat must be extracted from a large moist enthalpy reservoir (e.g., the ocean surface) and release heat after adiabatic expansion to a low moist enthalpy reservoir (e.g., the upper troposphere and lower stratosphere). As air spirals inward toward the center of low pressure, it undergoes near isothermal expansion, gaining moist enthalpy from the underlying surface. In other words, intensification is a function of the thermodynamic disequilibrium between the surface and the overlying near-surface atmosphere. Over the ocean, this heating is supplied by enthalpy fluxes from warm surface water, and air spirals inward isothermally. Also, warm surface water also provides an extensive source of water vapor which is essential for maintaining the strong convection in the region of lowest surface pressure. Even in the absence of significant wind shear, tropical cyclones generally decay as they migrate over land (or colder water). This is in response to a reduction in both heat input required to counteract adiabatic cooling and loss of the moisture supply or moist enthalpy for fueling deep convection^4^.

## The “Brown Ocean Effect”

Occasionally, a “Brown Ocean effect” can contribute to the intensification of tropical cyclones over land^5–9^. The Brown Ocean effect refers to saturated soils, swamps, and wetlands in the inland regions providing a source of moist enthalpy for maintaining tropical cyclone warm-core structures and inland intensification^6,8,10^. Thus, realistic representation of surface enthalpy fluxes is important for accurate model predictions of tropical disturbances over land^11^.

Several prior modeling and observational studies attest to the role of the Brown Ocean effect contributing to the unexpected intensification of tropical cyclones over land^5,6,8,12–15^. Wetter soil conditions are found to favor formation of mesoscale convection along with landfalling systems in coastal regions^16,17^. Tropical cyclones moving inland over northern Australia are occasionally observed to reintensify through process pathways other than classical extratropical rejuvenation^5,18^. These storms retain their warm-core structure, often redeveloping such features as eyes, and it is hypothesized that the revival is made possible by large vertical heat fluxes from a deep layer of very hot, sandy soil^13^. Increases in thermal diffusivity due to sandy soil wetted by the first rains from the approaching systems enable rapid upward diffusion of heat through the soil column, which is required to sustain warm-core storms of marginal hurricane intensity^10^. This intensification process is not unique to Australia; recent studies suggest that antecedent wet soils in the Indian monsoon region^16,19^, as well as the southeastern US and the US Southern Great Plains, have helped create an atmosphere conducive to tropical cyclone maintenance post-landfall, by enhancing surface latent heat fluxes^15,20^.

Over southern Louisiana, the moist landscape and Brown Ocean-like conditions pre-existed with swamps, wetlands and saturated soils; we hypothesize that the above discussed tropical storm sustenance conditions occurred in southern Louisiana and contributed to the intensification of flooding during the August 2016 event. This hypothesis is tested using numerical modeling experiments to assess the role of the Brown Ocean effect, namely that the land surface functions as a reservoir of moist enthalpy, which contributed to the development of the tropical disturbance into a persistent depression and very heavy rain over Louisiana during the period of 11–16 August 2016. Since it was located in southern parts of Louisiana, advection of warm, moist air from the south (Gulf of Mexico) further contributed to the sustenance of the system^1^.

## Methodology

We used the Weather Research and Forecasting (WRF 3.8.1) modeling system for conducting the numerical weather prediction (NWP) experiments to test the hypothesis on the impact of the Brown Ocean effect on the August 2016 Louisiana flooding event^21^. A grid with 3 km spacing over southern Louisiana and centered over the region most impacted by the flood was used in the WRF NWP modeling experiments, with the domain including all Gulf coast states and a considerable portion of the Gulf of Mexico. Figure 1b shows the entirety of this 3 km grid, overlaid with 850 mb geopotential height and wind barbs. The National Centers for Environmental Prediction (NCEP) Global Forecasting System (GFS) atmospheric analysis and forecast were used to initialize atmospheric conditions in the numerical model grids and also provide time-varying lateral boundary forcing. To initialize land surface conditions, we incorporated output from the NASA Short-term Prediction Research and Transition (SPoRT) Center Land Information System (LIS) assimilating Soil Moisture Active Passive (SMAP) data (Fig. 1c). The SPoRT-LIS^22,23^ runs the Unified Noah land surface model^24^ in an offline mode (i.e., uncoupled to an NWP model), forced by hourly meteorological analyses from the North American Land Data Assimilation System-2 (NLDAS-2)^25^ to produce observation-driven soil moisture and temperature analyses over the Continental US at ~3 km grid spacing. Using these best available estimates for soil initial conditions and land surface characteristics, WRF was used to simulate atmospheric evolution for a period of 8 days from 1200 UTC 8 August to 1200 UTC 16 August of 2016. Analysis nudging was applied above the boundary layer for the first 72 hours to establish the precursor meteorological conditions that led to the development of the tropical depression. Analysis nudging was discontinued after this period to minimize damping of small-scale processes resolved by experiments and the associated internal variability. Optimal model configuration (Table S1) used in the experiments was identified using an ensemble of simulations that considered multiple combinations of initial conditions, lateral boundary forcing and physical parameterizations (Table S2, Figure S3).

We then utilized the above-described simulation as the control and compare it against three sensitivity experiments that consider a combination of soil moisture, and land use and land cover (LULC) change scenarios that modify the potential Brown Ocean effect. These scenarios differ from the control experiments only in the soil moisture initial conditions and land cover classification over southern Louisiana. They are varied (Figure S1) as follows: 1) All wetlands in southern Louisiana are converted to open water; 2) All wetlands are converted to a cropland-natural vegetation mosaic and; 3) Same as scenario (2), except that the initial soil moisture in all the soil layers are reduced by 50%, which is similar to drier antecedent soil moisture conditions in the surrounding regions (Fig. 1c; western Louisiana and coastal areas of Mississippi). These LULC change simulations will be referred to herein as open water, cropland wet, and cropland dry experiments, respectively.

Note that the experimental design used in this study is different from prior studies that focused on inland intensification of tropical systems by conducting soil moisture sensitivity analysis^26^. Our experimental design considers variations in surface moisture availability from the perspective of land cover changes occurring in the region and its potential to impact similar events in the future. Analysis of satellite observations between 1985–2010 found the wetland loss rate to be ~43 km^2^ per year, which is equivalent to losing the area of a football field every hour^27^. The majority of the conversion is to cultivation, grassland, pasture/hay, developed open space, shrubland, urban development (low, medium, and high intensity), and to open water. The cropland vegetation mosaic is chosen as representative of wetland conversion due to anthropogenic activities. Conversion of natural wetlands to anthropogenic land use generally leads to a reduction in moisture availability, both due to changes in surface hydrology and land-atmosphere interactions resulting in rainfall reduction^28^. The cropland wet and cropland dry experiments represent extremes of surface moisture availability that could be expected for the anthropogenic land cover scenario applicable to this region.

Conversion of wetlands to open water is another transition that is important. Land cover change projections suggest transformation of 1300 km^2^ of wetlands to open water in coming years as a result of sea-level rise, land subsidence, and development^29^. This type of land cover change will result in a persistent source of surface moisture availability rather than that caused by chance occurrences of antecedent precipitation.

We hypothesize that the high soil moisture conditions (Brown Ocean) resulted in higher storm intensities and thus higher maximum wind speeds and lower minimum pressure. We also postulate that surface characteristics will be influential to the Brown Ocean effect, as they will determine the efficiency of moisture exchange/transport from the land to the atmosphere during storm intensification. The control and cropland wet experiments are scenarios where soil moisture is high, but the efficiency of moisture transport to the atmosphere is expected to vary due to differences in surface characteristics. Compared to the control scenario, moisture fluxes in the cropland dry scenario are affected due to differences in both soil moisture and surface characteristics. The open water scenario is expected to have features more akin to tropical low-pressure system intensification over the ocean.

## Results

**Figure 2:**
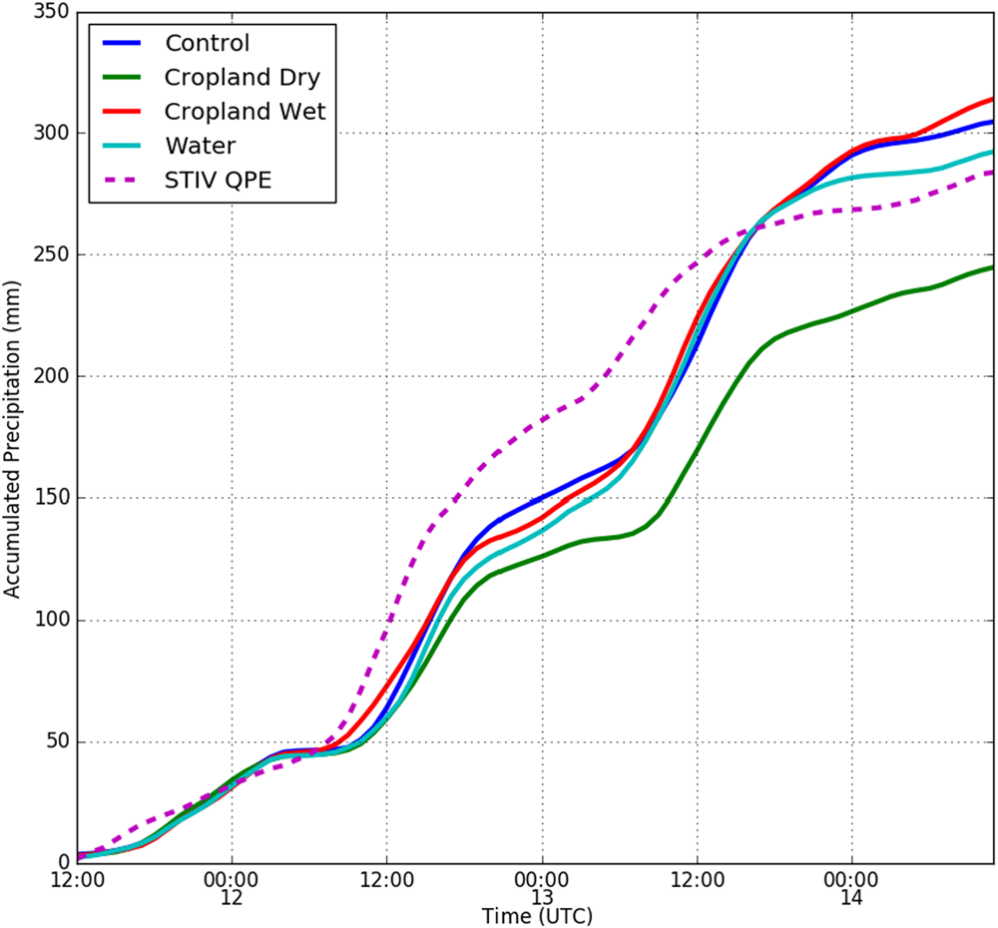
Rainfall for the control, cropland dry, cropland wet, and open water experiments, averaged over the 2° × 2° region centered on Baton Rouge (see Fig. 1c) are shown using red, yellow, green, and blue curves respectively. The purple dashed line shows the NCEP Stage IV hourly Quantitative Precipitation Estimate averaged over the same region.

Before examining the role of the Brown Ocean effect in these NWP experiments, we compared the hourly accumulated rainfall from the control experiment averaged over the 2° × 2° region centered on Baton Rouge (all area averages discussed from this point on in the manuscript are for this region) against the corresponding average of National Center for Environmental Prediction (NCEP) hourly Stage IV quantitative precipitation estimates (QPE, Fig. 2). Highest observed rainfall rates occurred between 0600 12 August-1800 UTC of 13 August and this pattern is well captured by the control simulation. However, the initial occurrence of high rainfall rates in the NWP experiments is delayed by ~3 hours compared to the observations. The average accumulated rainfall in the control experiment is 304.65 mm while the observed value was 283.80 mm. Point comparison against rain gauge observations at Baton Rouge also agrees well with the control experiment (Fig. S4).

**Figure 3:**
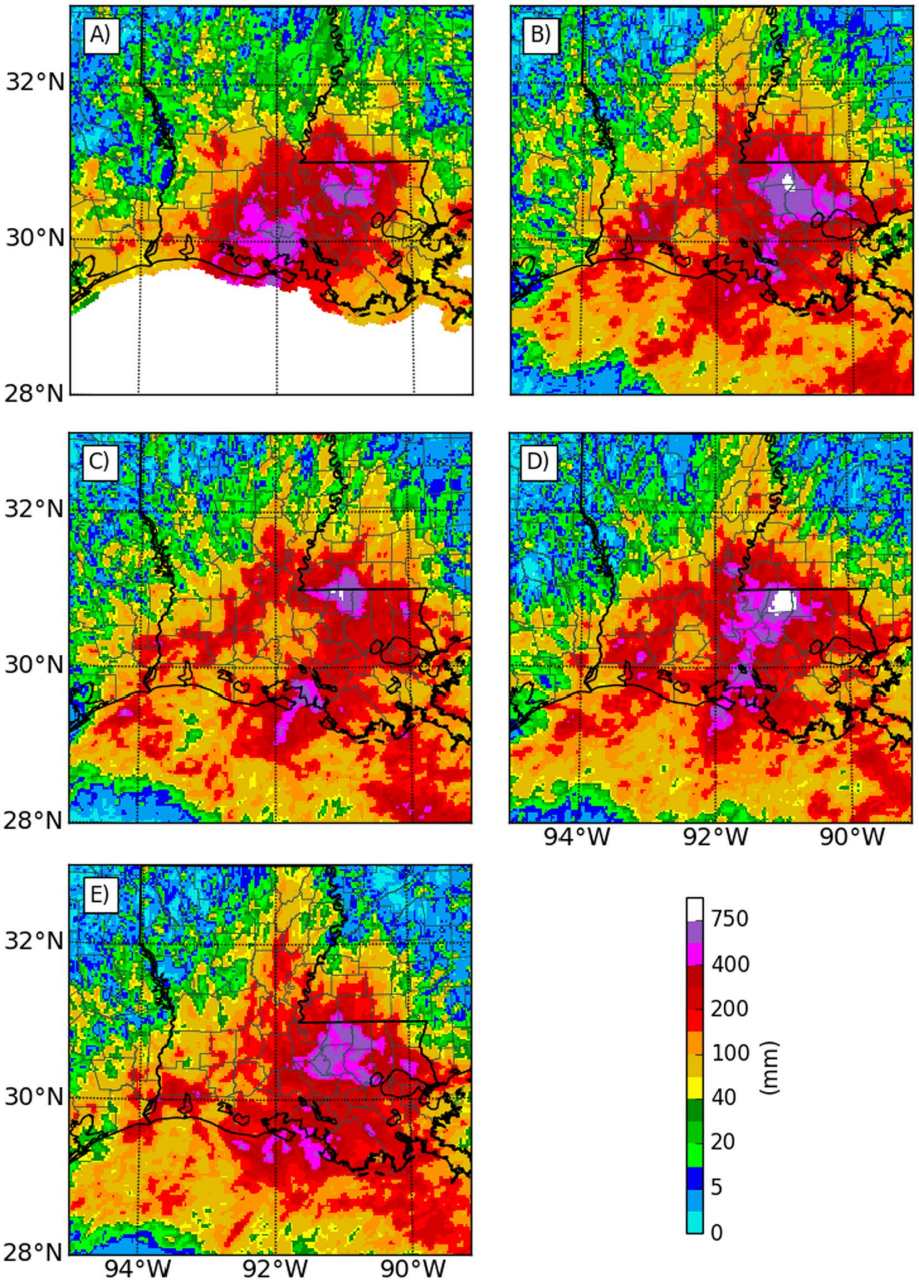
Spatial distribution of accumulated rainfall from: (**A**) Observations, (**B**) Control experiment, (**C**) Cropland dry experiment, (**D**) Cropland wet experiment and, (**E**) Open water experiment. Maps were created using Matplotlib, version 1.5.3^36^.

When compared to spatial patterns of NWS rainfall analysis, control experiments underestimate observed rainfall extremes over parishes to the southwest of Baton Rouge, including the Acadia, Iberia, Vermillion, and Lafayette parishes (Fig. 3A,B). However, the control experiment captures observed extreme rainfall accumulations in the vicinity of the study area, namely South Baton Rouge and Livingston parishes. The spatial pattern of rainfall accumulations in the control experiment is also consistent with other, prior numerical modeling studies of this event^1^. Thus, there is confidence in the skill of the control simulation to replicate the actual observed weather event. It is worth noting that there are negligible differences in the synoptic features of the simulations; the simulations broadly vary only by the storm strength itself.

**Figure 4:**
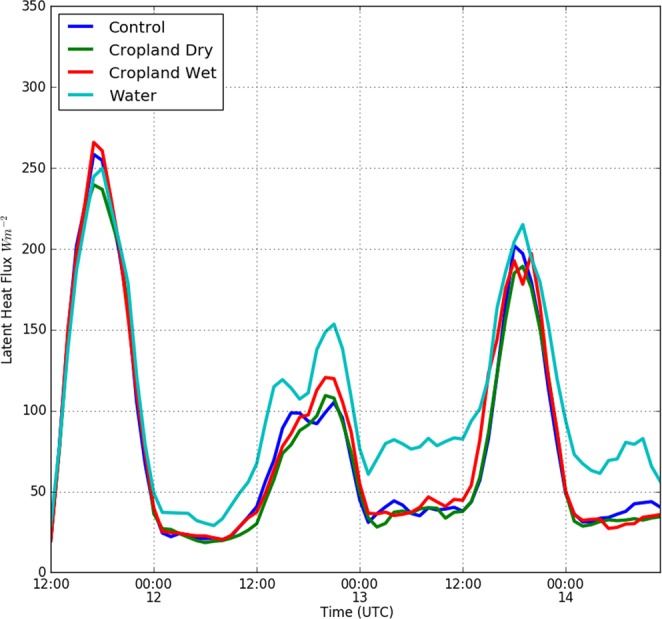
Surface latent heat fluxes for the control, cropland dry, cropland wet, and open water experiments, averaged over the 2° × 2° region centered on Baton Rouge (see Fig. 1c) are shown using blue, green, red, and aqua curves respectively.

**Figure 5:**
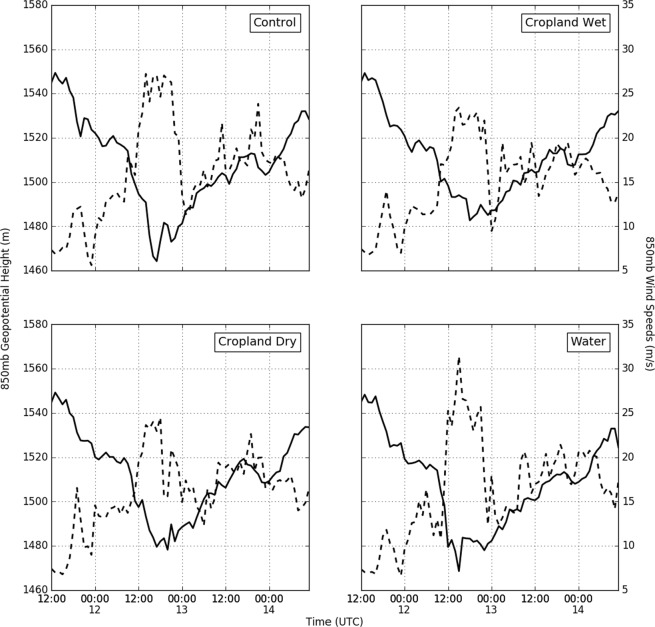
Time evolution of maximum wind speed (dashed) and minimum geopotential height at 850 hPa for the different experiments.

To examine the impact of the Brown Ocean effect and LULC change on storm structure, we analyzed the time evolution of area-averaged latent heat fluxes at the surface (Fig. 4), and minimum geopotential height and maximum wind speed (Fig. 5) at the 850 hPa level (Fig. 5). During daytime hours on 11 August, the experiments show the most substantial differences in area-averaged latent heat fluxes during the hours when surface insolation is high. This suggests that local buoyancy production of turbulent eddies is the dominant process driving moisture transport during this period. Latent heat fluxes are generally higher for the control, cropland wet and open water experiments compared to the cropland dry experiment during the daytime hours of the event. The highest average latent heat fluxes are found in the cropland wet experiment followed by the control, open water, and cropland dry experiments.

However, as the storm starts to intensify during the early hours of 12 August (Fig. 5), the highest average latent heat fluxes occur in the open water experiment followed by the control, cropland wet, and cropland dry experiments. Differences in latent heat fluxes for the entire event time period are statistically significant only for comparisons between open water and other experiments.

The highest maximum wind speed occurs in the open water experiment (31.40 ms^−1^), followed by the control (27.22 ms^−1^), cropland dry (24.50 ms^−1^), and cropland wet (23.41 ms^−1^) experiments during the intensification stage. The minimum 850 hPa geopotential height is lowest in the control experiment (1464.3 m), followed by the open water (1468.6 m), cropland dry (1478.3 m), and cropland wet (1482.7 m) experiments. Thus, a negative correlation between a minimum of 850 hPa geopotential heights and maximum winds is found only for a subset of the experiments. However, such a pattern is not unusual for small tropical systems^30^.

Our experiments show that, as expected, the storm achieves maximum intensity in the open water experiment; and it was interesting to note that storm intensification is adversely affected when the land cover is converted to croplands (Fig. 5). Whereas the cropland wet experiment had slightly higher 850 hPa minimum geopotential height and lower maximum wind speeds compared to the cropland dry experiment, higher intensity conditions were maintained in the former scenario for a substantially longer period compared to the latter scenario. Consistent with the Brown Ocean hypothesis, intensification of the storm is clearly impacted by the moisture availability at the surface. However, the Brown Ocean Effect is also shown to be sensitive to the nature of the land cover, as changes in roughness modulate heat, moisture, and momentum transfer to the atmosphere. Interactively these changes affect the mesoscale convection and rainfall resulting from the storm.

LULC change scenarios considered in the experiments cause statistically significant differences in average rainfall accumulations over the Baton Rouge area (Fig. 2). The time evolution of area-averaged rainfall in all the experiments with high initial surface moisture availability (control, open water, and cropland wet) is similar during the two convective pulse events with high rainfall rates but diverges after the cessation of such events. Compared to the control experiment, the cropland wet and open water experiments resulted in differences in average accumulated rainfall of +3% and −4% respectively, at the end of the analysis period considered. The rainfall evolution in the cropland dry experiment shows substantial differences compared to the other experiments. The rain rates during the two convective pulse events are lower in the cropland dry experiment and lead to a 20% reduction of area-averaged rainfall at the end of the analysis period (Fig. 2).

The spatial distribution patterns of rainfall from the storm also show statistically significant differences between the experiments. The main region of enhanced rain (defined here as >400 mm) in the cropland wet experiment (Fig. 3D) is similar to the control scenario (Fig. 3B), but the southern portions are more extensive in the cropland wet experiment. The area of enhanced rainfall in the cropland wet experiment is greater (11,331 km^2^) compared to the control experiment (9,342 km^2^). Also, the area with accumulated rainfall exceeding 750 mm (most of East Feliciana parish) is also substantially higher in the cropland wet experiment (937 km^2^) compared to the control experiment (306 km^2^). The region of enhanced precipitation in the open water experiment is reduced (8586 km^2^) compared to both control and cropland wet experiments, and the least extent is found in the cropland dry experiment (4,689 km^2^). The area where accumulated rainfall exceeds 750 mm is 99 km^2^ in the cropland dry experiment, whereas none is present in the open water experiment.

Note that the direct effect of Brown Ocean, which is inland enhancement of latent heat fluxes and convection (Figure S5), is not the only reason for intensification of the storm. Intensification resulting from the direct effect can also indirectly cause additional impact through increase in radial moisture transport from the ocean. We examined the role of this indirect effect by conducting back trajectory analysis for ~1400 locations over the Baton Rouge area, starting at 12 UTC on the 13 August and extending back for a period of 24 hours. Mean values of air parcel properties and position was computed for the trajectories at regular intervals times (Figures S6 and S7). On average, latent heat fluxes and wind speeds increase as these trajectories approach land and are substantially higher in the cropland wet and open water experiments, with the enhancement extending some distance inland. Radius-azimuth plots of moisture transport (Figure S8) do indeed show increased southerly transport of moisture in the control, open water, and cropland wet experiments compared to cropland dry experiments. However, there are also substantial contributions to moisture transport from inland regions and directions other than southerly. These components of moisture transport do respond to changes in land cover and soil moisture and are enhanced in the open water and cropland wet experiments.

Also, changes in surface roughness with land cover also contribute to differences in storm evolution between the experiments^31–33^. A prior study^33^, utilizing idealized numerical modeling, examined how intensity and rainfall patterns of landfalling hurricanes respond to variations of moisture availability and surface roughness. In these experiments, rainfall maximums are found on the right side of the approaching storm, with drier land surfaces enhancing this rainfall asymmetry. This is caused by destabilization induced by lower equivalent potential temperature air from land, circulating cyclonically and intruding above surface air on the rear right-hand side of the storm. Upon landfall, rainfall maximum switches to the left side of the storm, with frictionally-driven convergence of the radial component of the storm circulation playing an important role in forcing this feature. On the right-hand side of the storm, speed convergence of the tangential wind component occur as the offshore flow encounters a land surface with higher surface roughness compared to the ocean surface. While convection is also enhanced in this region, advection of rainfall downwind further contributes to rainfall maximum on the left side of the storm. Idealized experiments also examined how landfalling hurricanes respond to changes in surface roughness while keeping the moisture availability constant and vice versa. These experiments found that decay of landfalling hurricanes is more sensitive to an increase in surface roughness than to a decrease in moisture availability. This is attributed to a decrease in surface wind speeds caused by higher surface roughness thereby reducing surface latent and sensible heat fluxes.

Even though the nature of the storm (near stationary vs. land falling) and experiments conducted (homogenous vs. heterogeneous surface characteristics) are substantially different in the present study, some aspects of the above-described findings from idealized numerical modeling experiments provide a conceptual basis for analyzing differences found in the LULC experiments. The storm remained relatively stationary during most of its duration, straddling the Louisiana coast. As seen in the idealized experiment for the landfalling stage, end members of the experiments in the current study show that rainfall distribution is most impacted by moisture availability (least in the dry cropland experiments) while surface roughness has the highest impact on storm intensity (maximum in the open water experiment). In the open water experiment, rainfall on the right side of the storm increases in areas near the coast that were converted to open water (Fig. 3E), consistent with rainfall enhancement expected over open water on the onshore flow side of the storm. Rainfall over the open ocean in the offshore flow side of the storm is also enhanced in the dry cropland experiment (Fig. 3C,D).

Within the four quadrants of a 2° × 2° region centered over the storm (Figure S9), we also examined time evolution of average wind speed (Figure S10), wind direction (Figure S11), and rainfall (Figure S12). Maximum differences in the wind speed are found in the lower left and right quadrants. The open water experiment, with the least mean roughness in these quadrants, also has the highest wind speeds. The next highest wind speeds in these quadrants are found in the cropland wet experiment, which also has the next lowest mean roughness. The control experiment, with the highest mean roughness in the lower left and right quadrants, generally shows wind speeds that are smaller compared to both the open water and cropland wet experiment.

During the intensification phase of the storm, maximum differences in rainfall between the experiments occur in the lower right quadrant, with the control and open water experiment having substantially higher rainfall compared to the other experiments. Note that the changes in surface characteristics between the LULC experiments are also maximized in the lower right quadrant (Figure S9). In the open water experiment, enhancement of rainfall results from higher radial moisture transport resulting from increased flow speeds. However, rainfall in the lower right quadrant is highest in the control experiment, which appears to be caused by enhanced radial transport caused by a directional change in the wind due to higher roughness. In the cropland wet experiment, less radial moisture transport in the lower right quadrant occurs during the intensification phase as wind speed is reduced compared to the open water experiment and is not compensated by directional changes as in the control experiment. Concerning the above discussions, note that changes in moisture transport caused by frictional effects feedback on wind fields through latent heat release and associated alteration of pressure fields. Reduced rainfall in the lower right quadrant in the cropland wet experiment results in high moisture transport to other quadrants and combined with higher wind speeds results in high rainfall in the upper right and left quadrants in this experiment. Differences between the cropland wet and dry experiments suggest that both the reduction in rainfall and storm intensity are driven by diminished moisture availability.

## Conclusions

Synthesis of the numerical modeling results shows that the tropical system that caused the August 2016 extreme Louisiana flooding event is indeed sensitive to the Brown Ocean effect. For scenarios where the total land area was not modified, the existing distribution of wetlands combined with high antecedent soil moisture conditions leads to storm intensification that most closely resembles the intensification pattern expected over oceans. Wetlands to cropland transitions resulted in reduction of storm intensity irrespective of soil moisture conditions. Drier conditions also caused a 20% reduction in rainfall and shorter durations of high wind conditions. Conversion of wetlands to open water, where the total land area was reduced, resulted in the highest intensity storm. In addition, areal redistribution of rainfall also occurred, reducing rainfall over Baton Rouge while increasing it over areas upwind.

We also found that the modulation of the Brown Ocean effect by land cover change primarily occurs through processes linked to alterations in wind speed and direction. Near-surface wind speeds and direction are both affected by surface roughness. The open water experiment has the lowest surface roughness and maximum onshore wind speed, which in conjunction with high surface moisture availability and moisture cause the formation of the highest intensity storm. Maximum surface roughness in the coastal regions occurs in the control experiment reducing onshore wind speeds. However, cross-isobaric flow is enhanced and when combined with high moisture availability, partially compensates for reduced wind speeds. In the cropland wet experiment, where surface roughness is reduced compared to the control experiment while keeping soil moisture constant, an increase in onshore flow is not sufficient to compensate for decreased cross-isobaric flow. This leads to moisture transport further inland and a localized increase in rainfall. Differences between the cropland wet and dry simulations shows that after controlling surface roughness effects, both storm intensity and rainfall reduce in response to lower moisture availability.

Prior studies^26^ on the intensification of tropical lows over land found that horizontal moisture transport into the system is approximately equivalent to moisture loss through rainfall. Further, they found that the contribution of surface fluxes of moisture to the total water budget to be small, but important to modulation of convection in the vicinity of the circulation center. The present study suggests that this also applies to the 2016 Louisiana event and drier conditions would have led to reduced storm intensity, substantial reduction of moisture transport within the storm, and hence the drastic reduction in accumulated precipitation.

The two major land surface transitions considered in this study, namely conversion of wetlands to cropland mosaic and open water, are reflective of changes that have occurred and continue to occur in southern Louisiana. Our experiments show that conversion of wetlands to cultivated land will weaken tropical systems such as the one that caused the 2016 Louisiana flood, with the degree of weakening controlled by antecedent soil moisture. On the other hand, conversion of wetlands to open water will lead to intensification of the system and redistribution of rainfall from such events, but persisting in quantities still capable of causing flooding. In other words, the wetland restoration efforts could have broader implications for the region’s resiliency.

It has been suggested that the probability of tropical system-midlatitude interactions that provided forcing for the mid-August 2016 Louisiana floods^1^ has been enhanced due to more frequent propagation of (potentially stronger) upper-level troughs from the western US to the Gulf Coast. Combined with a projected increase in precipitable water due to anthropogenic climate warming, the return time of an event such as the mid-August Louisiana flood event is expected to decrease^34^. Our studies suggest that local LULC change is also of importance. If the current trend continues, LULC change studies in this region indicate that a substantial portion of the wetlands will transition to open water in the coming decades. This will add another ingredient, namely a persistent source of surface moisture availability, i.e., the Brown Ocean Effect will favor recurrence of events such as the 2016 Louisiana floods. Continued LULC transition to open water would likely make the region even more vulnerable to heavy rain events from future tropical systems.

## Supplementary information

Supplementary material

## Data Availability

The datasets generated during the current study are available from the corresponding author on reasonable request.

## References

[1] Wang S.-Y. Simon, Zhao Lin, Gillies Robert R. (2016). Synoptic and quantitative attributions of the extreme precipitation leading to the August 2016 Louisiana flood. Geophysical Research Letters.

[2] Terrell, D. *The Economic Impact of the August 2016 Floods on the State of Louisiana*. (2016).

[3] Emanuel K (1991). The Theory Of Hurricanes. Annu. Rev. Fluid Mech..

[4] Pielke, R. A. Jr. & Pielke, R. A. Sr. *Hurricanes: Their nature and impacts on society*. (John Wiley and Sons, England, 1997).

[5] Emanuel K, Callaghan J, Otto P (2008). A Hypothesis for the Redevelopment of Warm-Core Cyclones over Northern Australia. Mon. Weather Rev..

[6] Chang HI (2009). Possible relation between land surface feedback and the post-landfall structure of monsoon depressions. Geophys. Res. Lett..

[7] Bozeman, M. L. *et al*. An HWRF-based ensemble assessment of the land surface feedback on the post-landfall intensification of Tropical Storm Fay (2008). *Natural Hazards* **63** (2012).

[8] Andersen TK, Shepherd JM (2014). A global spatiotemporal analysis of inland tropical cyclone maintenance or intensification. Int. J. Climatol..

[9] Andersen Theresa, Shepherd Marshall (2017). Inland Tropical Cyclones and the “Brown Ocean” Concept. Hurricanes and Climate Change.

[10] Kishtawal CM, Niyogi D, Kumar A, Bozeman ML, Kellner O (2012). Sensitivity of inland decay of North Atlantic tropical cyclones to soil parameters. Nat. Hazards.

[11] Simpson, R. H. & Pielke, R. A. Hurricane development and movement. *Appl. Mech. Rev*. **29** (1976).

[12] Dastoor A, Krishnamurti TN (1991). The landfall and structure of a tropical cyclone: The sensitivity of model predictions to soil moisture parameterizations. Boundary-Layer-Meteorology.

[13] Kellner, O., Niyogi, D., Lei, M. & Kumar, A. The role of anomalous soil moisture on the inland reintensification of Tropical Storm Erin (2007). *Nat. Hazards* **63**, 1573–1600 (2012).

[14] Andersen TK, Radcliffe DE, Shepherd JM (2013). Quantifying surface energy fluxes in the vicinity of inland-tracking tropical cyclones. J. Appl. Meteorol. Climatol..

[15] Evans C, Schumacher RS, Galarneau TJ (2011). Sensitivity in the overland reintensification of Tropical Cyclone Erin (2007) to near-surface soil moisture characteristics. Mon. Weather Rev..

[16] Kishtawal CM (2013). Enhancement of inland penetration of monsoon depressions in the Bay of Bengal due to prestorm ground wetness. Water Resour. Res..

[17] Xian, Z. A 2-D model study of the influence of the surface on mesoscale convection during the Indian monsoon. *Department of Atmospheric Science*. (Colorado State University, 1991).

[18] McGrath GS (2012). Tropical cyclones and the ecohydrology of Australia’s recent continental-scale drought. Geophys. Res. Lett..

[19] Niyogi, D. *et al*. In *Land -Atmospheric Interactions in Asia* (eds Vadrevu, K. P., Ohara, T. & Justice, C.) (Springer, 2017).

[20] Monteverdi JP, Edwards R (2010). The Redevelopment of a Warm-Core Structure in Erin: A Case of Inland Tropical Storm Formation. E-Journal Sev. Storms Meteorol..

[21] Skamarock, W. C. *et al*. A Description of the Advanced Research WRF Version 3. *Tech. Rep*. **113**, 10.5065/D6DZ069T (2008).

[22] Zavodsky, B. T., Case, J. L., Blankenship, C. B., Crosson, W. L. & White, K. D. Application of Next-Generation Satellite Data to a High-Resolution, Real-Time Land Surface Model | Earthzine. *Earthzine* 1–10 (2013).

[23] Case JL (2016). From drought to flooding in less than a week over South Carolina. Results Phys..

[24] Ek MB (2003). Implementation of Noah land surface model advances in the National Centers for Environmental Prediction operational mesoscale Eta model. J. Geophys. Res..

[25] Xia Youlong, Mitchell Kenneth, Ek Michael, Cosgrove Brian, Sheffield Justin, Luo Lifeng, Alonge Charles, Wei Helin, Meng Jesse, Livneh Ben, Duan Qingyun, Lohmann Dag (2012). Continental-scale water and energy flux analysis and validation for North American Land Data Assimilation System project phase 2 (NLDAS-2): 2. Validation of model-simulated streamflow. Journal of Geophysical Research: Atmospheres.

[26] Tang S, Smith RK, Montgomery MT, Gu M (2016). Numerical study of the spin-up of a tropical low over land during the Australian monsoon. Q. J. R. Meteorol. Soc..

[27] Couvillion, B. R. *et al*. *Land area change in coastal Louisiana (1932 to 2010)*. (2011).

[28] Marshall CH, Pielke RA, Steyaert LT, Willard DA (2004). The Impact of Anthropogenic Land-Cover Change on the Florida Peninsula Sea Breezes and Warm Season Sensible Weather. Mon. Weather Rev..

[29] Barras J (2004). Historical and Projected Coastal Louisiana Land Changes: 1978-2050. World.

[30] Callaghan J, Smith RK (1998). The relationship between maximum surface wind speeds and central pressure in tropical cyclones. Aust. Meteorol. Mag..

[31] Tuleya RE (1994). Tropical Storm Development and Decay: Sensitivity to Surface Boundary Conditions. Monthly Weather Review.

[32] Tuleya RE, Bender MA, Kurihara Y (2002). A Simulation Study of the Landfall of Tropical Cyclones. Monthly Weather Review.

[33] Kimball SK (2008). Structure and Evolution of Rainfall in Numerically Simulated Landfalling Hurricanes. Mon. Weather Rev..

[34] van der Wiel K (2016). Rapid attribution of the August 2016 flood-inducing extreme precipitation in south Louisiana to climate change. Hydrol. Earth Syst. Sci. Discuss..

[35] MCST. *MODIS Level 1B Product User’s Guide, Version 6.1.14 (Terra)/Version 6.1.17(Aqua)*. (2012).

[36] Hunter JD (2007). Matplotlib: A 2D Graphics Environment. Comput. Sci. Eng..

