# Supplementary material for: Influence of Land Cover and Soil Moisture based Brown Ocean Effect on an Extreme Rainfall Event from a Louisiana Gulf Coast Tropical System
Source: Sci Rep. 2019 Nov 20;9:17136. doi: 10.1038/s41598-019-53031-6 (PMC6868174; doi:10.1038/s41598-019-53031-6)
Supplement: Supplementary file 1 — Supplementary material [file 41598_2019_53031_MOESM1_ESM.docx]

**Supplementary material**

**Influence of Land Cover and Soil Moisture based Brown Ocean Effect on an Extreme Rainfall Event from a Louisiana Gulf Coast Tropical System**

**Udaysankar S. Nair^1^, Eric Rappin^2^, Emily Foshee^1^, Warren Smith^3,4^, Roger A. Pielke Sr.^4^, Rezaul Mahmood^5^, Jonathan L. Case^6^, Clay B. Blankenship^7^, Marshall Shepherd^8^, Joseph A. Santanello^9^, Dev Niyogi^10^**

**^1^Department of Atmospheric Science, University of Alabama in Huntsville, Huntsville, AL 35806**

**^2^ Department of Geography and Geology and Kentucky Climate Center, Western Kentucky University, Bowling Green, KY 42101**

**^3^Department of Atmospheric and Oceanic Sciences, University of Colorado Boulder, Boulder, CO 80309**

**^4^Cooperative Institute for Research in Environmental Sciences, University of Colorado Boulder, Boulder, CO 80309**

**^5^High Plains Regional Climate Center, School of Natural Resources, University of Nebraska-Lincoln, Lincoln, NE 68583**

**^6^ENSCO, Inc./NASA Short-term Prediction Research and Transition (SPoRT ) Center**

**^7^ Universities Space Research Association, NASA Short-term Prediction Research and Transition (SPoRT) Center**

**^8^University of Georgia, Department of Geography, Atmospheric Sciences Program**

**^9^NASA-GSFC, Hydrological Sciences Laboratory, Greenbelt, MD**

**^10^Department of Agronomy and Department of Earth, Atmospheric and Planetary Sciences, Purdue University, West Lafayette, IN 47907, USA**

**WRF Configuration:** The Weather Research and Forecast (WRF) model is a regional, non-hydrostatic numerical model developed by the National Center for Atmospheric Research ^20^. WRF solves governing equations of atmospheric flow utilizing finite difference formulations on an Arakawa C-Grid in the horizontal and terrain-following sigma-p coordinates in the vertical. A variety of parameterization schemes are available for representing physical processes, the details of which are provided in *Skamarock et al.* [2008].

In this study, the Advanced Research WRF model version 3.8.1 was configured using a single domain with a 3 km grid spacing in both the x and y directions centered at 30.5^0^N, 92^0^W. A total of 560x560 horizontal mass points and 61 vertical levels are utilized with the 15 lowest vertical levels being located within the planetary boundary layer. Details of the physical parameterizations and other relevant model configuration variables are provided in Table 1.

The Short-Term Prediction and Research Transition Center's (SPoRT) Real-Time Land Information System (LIS) provides high resolution land surface initial conditions for weather prediction models at real time. The SPoRT LIS domain of 3 km grid spacing covers most of the central and eastern United States and ingests NASA MODIS satellite-derived Greenness Vegetation Fraction and thus provides observational constraints on vegetation phenology. The land cover classification in WRF is specified using the MODIS land cover dataset (Figure S1).

| **Domain** | |
| --- | --- |
| Number of Domains | 1 |
| Grid Spacing (dx & dy) | 3 km |
| Start Date | August 8, 2016 12Z |
| End Date | August 16, 2016 12Z |
| Input Data | FNL & SPoRT-LIS land surface conditions |
| Center Latitude/Longitude | 30.5ºN, 92.0ºW |
| Input Data Interval | 6 hours |
| Number of Vertical Levels | 61 |
| Number of Vertical Levels in Lowest 1 km | 15 |
| Time Step | 15 seconds |
| Radiation Time Step | 5 minutes |
| Top Pressure Level | 5000 Pa |
| Number of Soil Layers | 4 |
| Static Geographic Data Resolution | 15 seconds |
| **Parameterization Schemes** | |
| Microphysics | Thompson aerosol-aware (option 8) |
| Radiation (Shortwave) | RRTMG (option 4) |
| Radiation (Longwave) | RRTMG (option 4) |
| Planetary Boundary Layer | Mellor-Yamada-Janjic (option 2) |
| Cumulus | None |
| Surface Layer | Monin-Obukhov (Janjic, option 2) |
| Land/Water Surface | Noah Land Surface Model (option 2) |
| Urban | None |
| **Dynamics** | |
| Turbulence/Mixing | W-Rayleigh (Relaxation, option 3) |
| Diffusion | 2^nd^ Order (option 1) |

**Table S1: WRF options used in each of the discussed simulations**


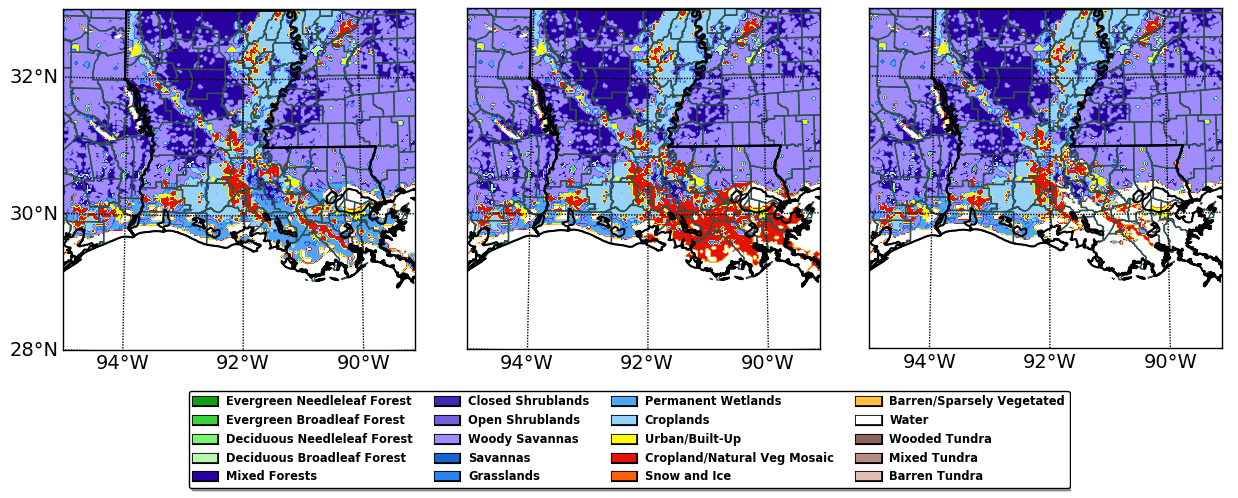


**Figure S1.** Land cover map applicable to the control, cropland wet, cropland dry, and open water experiments are shown in the top left, top right, bottom left, and bottom right panels, respectively. Maps were created using Matplotlib, version 1.5.3 (<https://matplotlib.org/1.5.3/contents.html>).


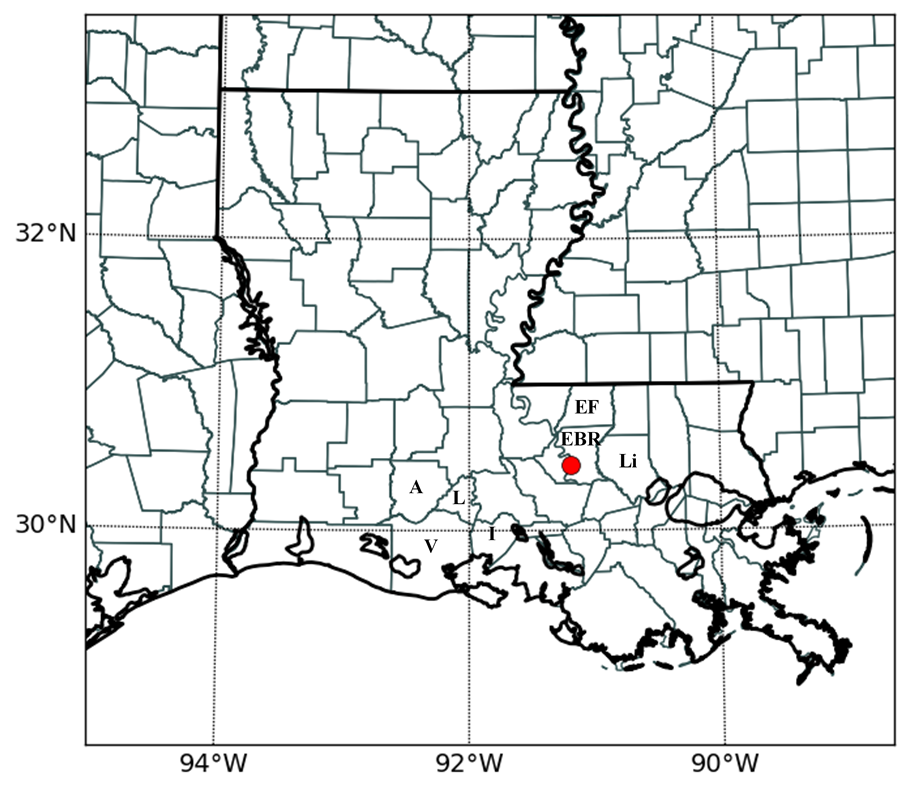


**Figure S2.** Locations of the East Feliciana, East Baton Rouge, Livingston, Acadia, Lafayette, Iberia, and Vermilion parishes are marked as EF, EBR, Li, A, L, I and V respectively. Maps were created using Matplotlib, version 1.5.3 (<https://matplotlib.org/1.5.3/contents.html>)

**Selection of model parameterization and initial/boundary conditions.** An ensemble of simulations were conducted (Table S2) to determine which suite of physical parameterizations and initial/boundary conditions accurately reproduced the magnitude and geographical distribution of precipitation for the flood event. As the lower boundary initial condition was generated with the Noah Land Surface Model, that parameterization was not changed. The table below provides information on.

| Experiment | Lateral Forcing | Surface Forcing | Microphysics | PBL physics |
| --- | --- | --- | --- | --- |
| Control | FNL | LIS SMAPDA | Thompson | MYJ |
| 1 | FNL | LIS | Thompson | MYJ |
| 2 | NAM | LIS SMAPDA | Thompson | MYJ |
| 3 | NAM | LIS | Thompson | MYJ |
| 4 | FNL | LIS SMAPDA | WSM6 | MYJ |
| 5 | FNL | LIS SMAPDA | Thompson | YSU |
| 6 | FNL | LIS SMAPDA | Thompson | MYNN2 |

**Table S2**. different initial/boundary conditions and parameterizations considered in the ensemble of simulations. FNL: NCEP Final Global Analysis; NAM: 12 km North American Mesoscale Forecast System Analysis; LIS: NASA SPoRT 3 km Land Information System; LIS SMAPDA: NASA SPORT 3 km LIS with SMAP Data Assimilation; Thompson: Thompson microphysics – double moment for ice and rain; WSM6: WRF single moment six class microphysics; MYJ: Mellor – Yamada – Janjic PBL scheme; YSU: Yonsei University PBL scheme; MYNN2: Mellor – Yamada – Nakanishi – Nino level 2.5 PBL scheme.

**a)** **b)**


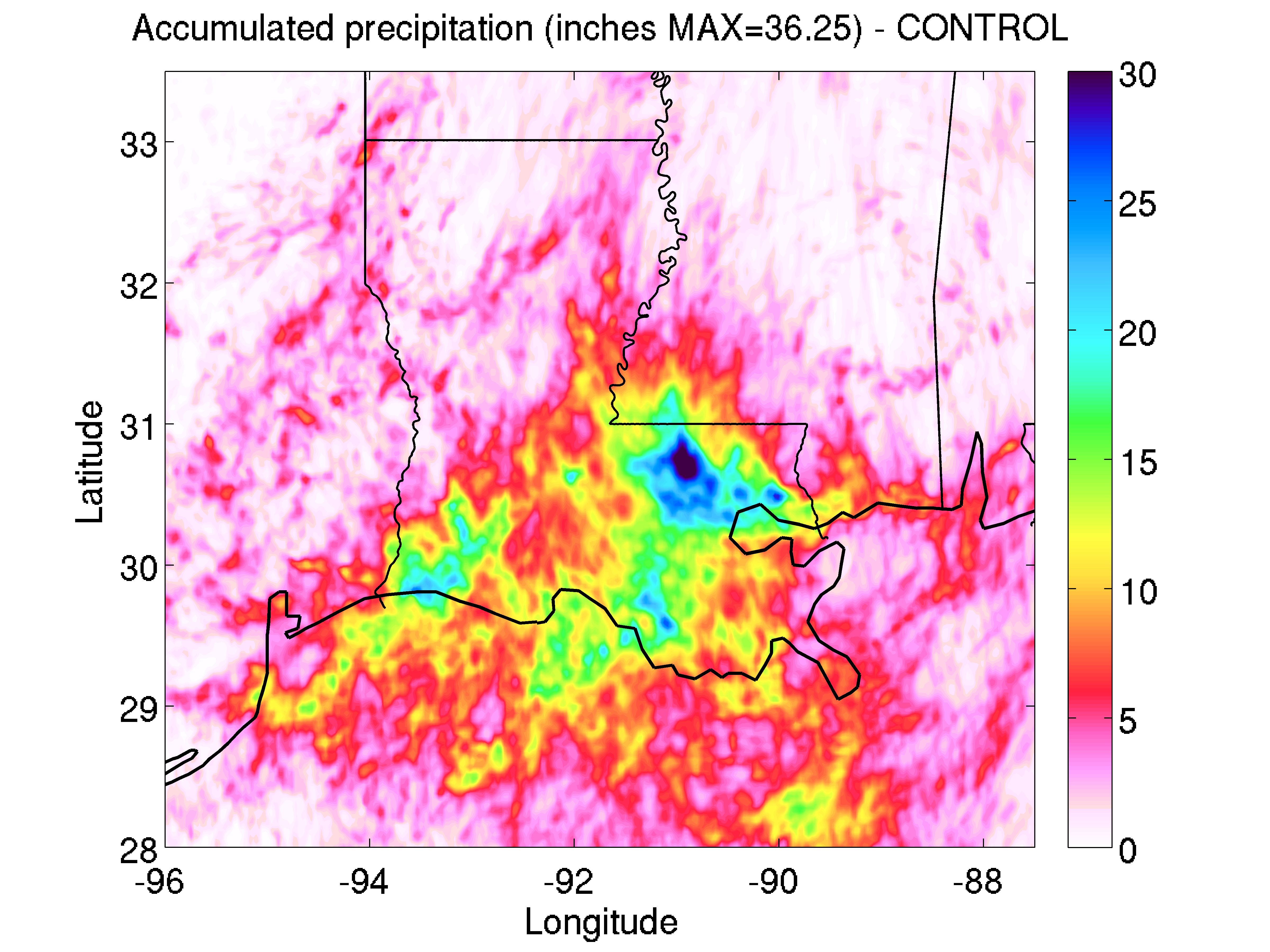

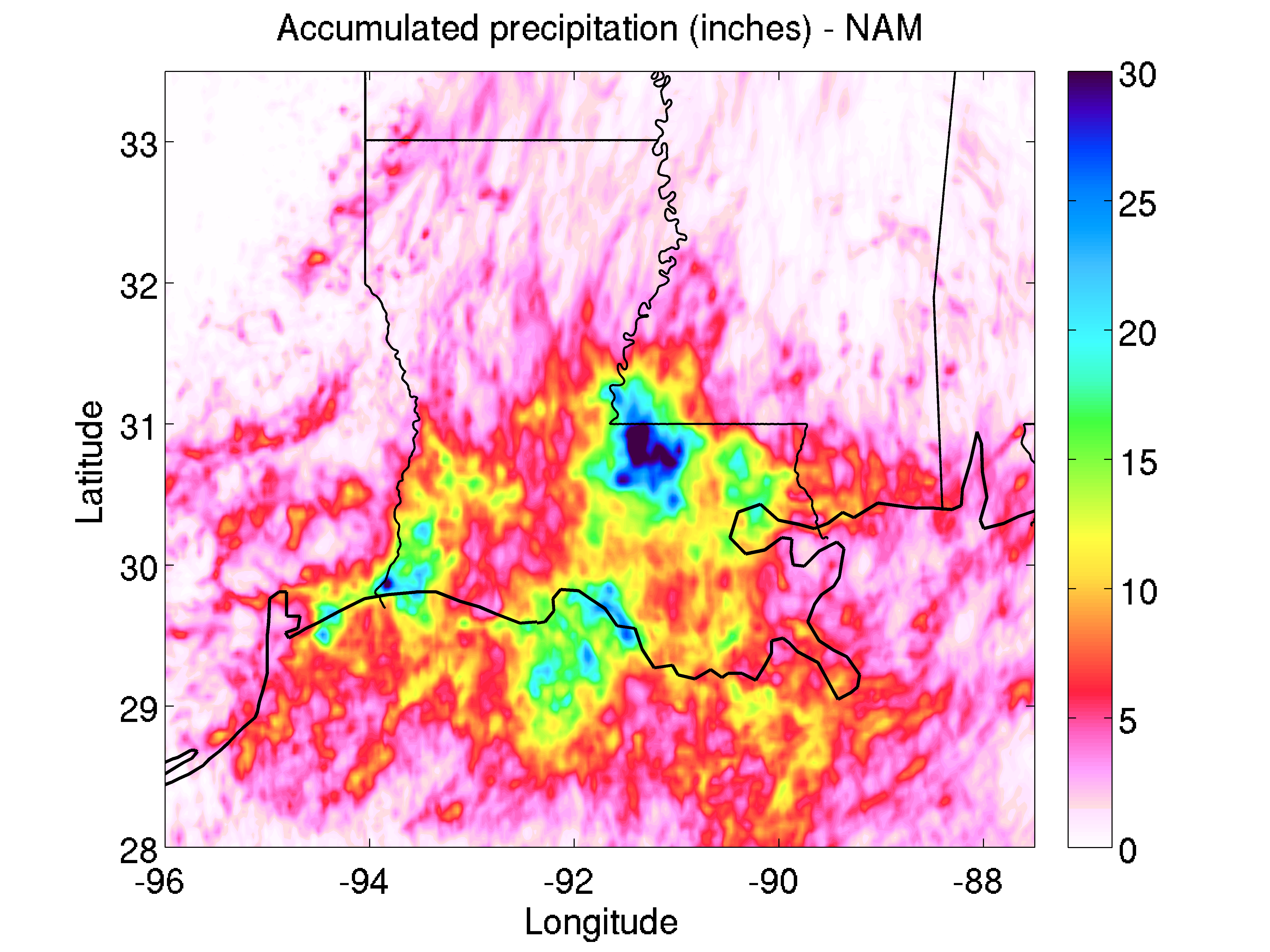


**c)** **d)**


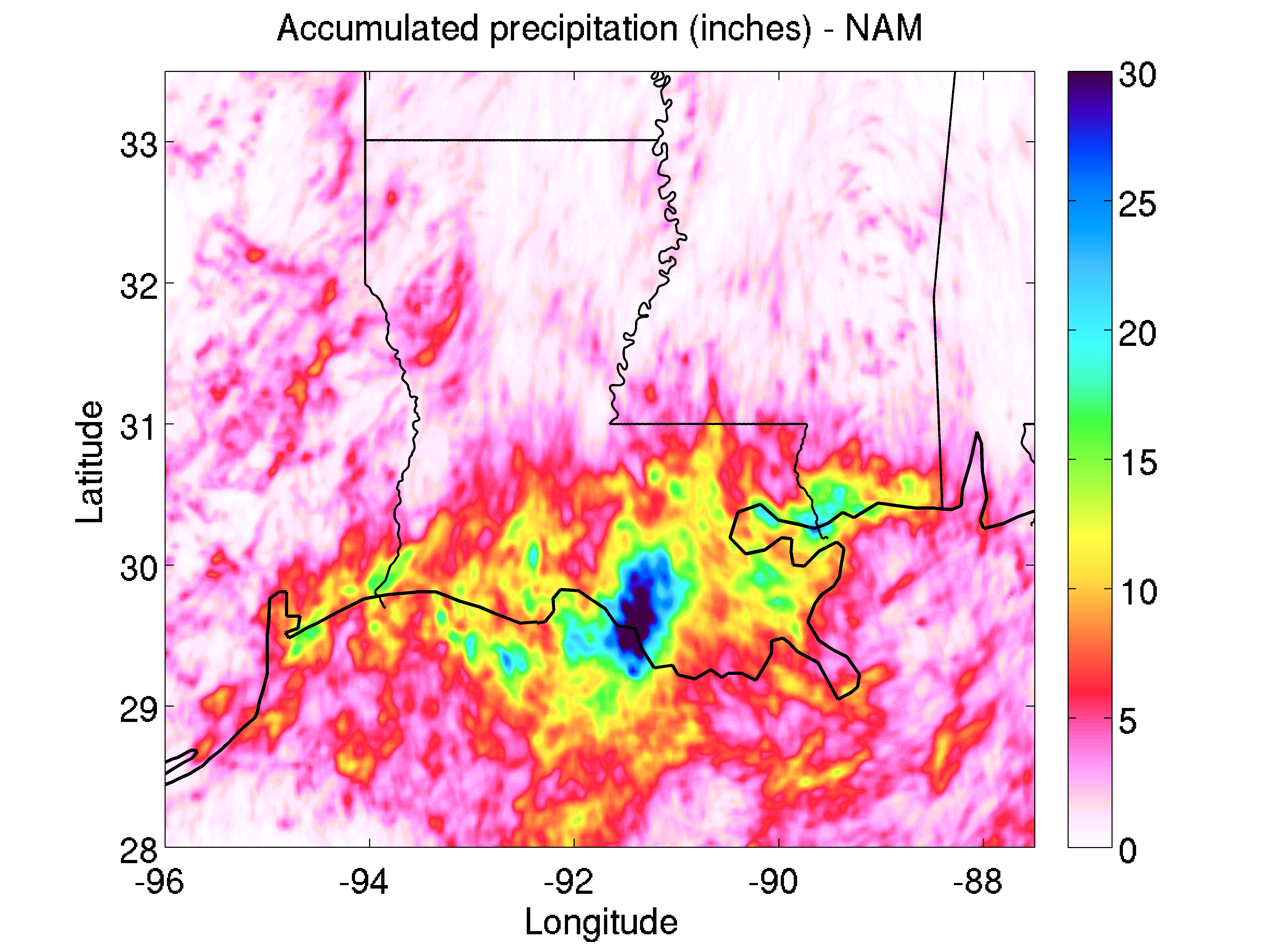

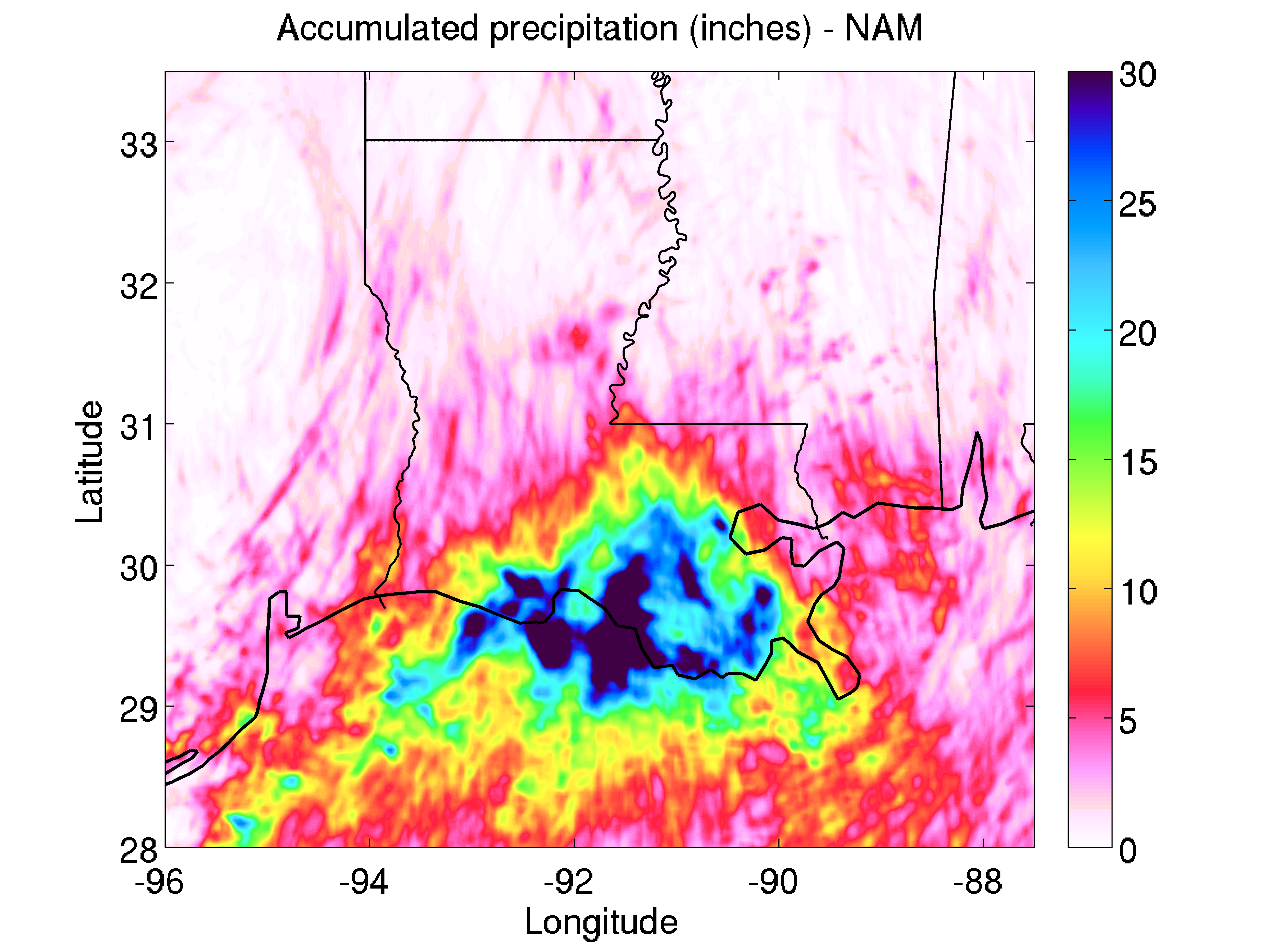


Physics and forcing ensemble performed: a) Control, b) Exp. 1, c) Exp. 2, d) Exp. 6.

Figure S3. Spatial patterns of accumulated precipitation (inches) from: a) Control; b) Exp 1, c) Exp 2 and, d) Exp 3. Maps were created using Matlab R2017a ( <https://www.mathworks.com/products/new_products/release2017a.html>)


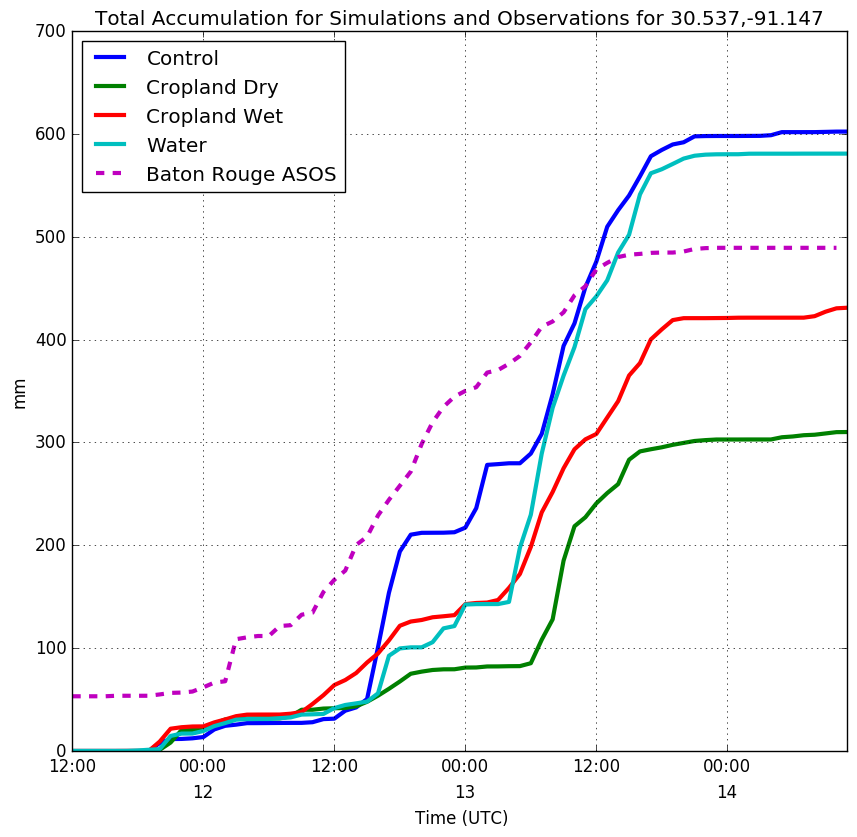


Figure **S4**. Model-simulated rainfall at the ASOS location showing a wide range of variability in response to soil moisture and LULC changes (Figure 2). All considered LULC change scenarios led to local reduction in rainfall at the ASOS observation location. Compared to the control simulation, the open water, cropland dry, and cropland wet LULC change scenarios resulted in local rainfall changes of -3%, -26%, and -48%, respectively.

**Analysis of Latent Heating:** Vertical profiles of latent heating for the experiments also show differences among the experiments (Figure S5). Prior to the intensification of the system, substantial differences in the vertical profile of latent heating is found, with the cropland wet experiment showing up to 50% more latent heating compared to other experiments. Note the initial decline in 850 hPa geopotential height is highest for the cropland wet experiment.


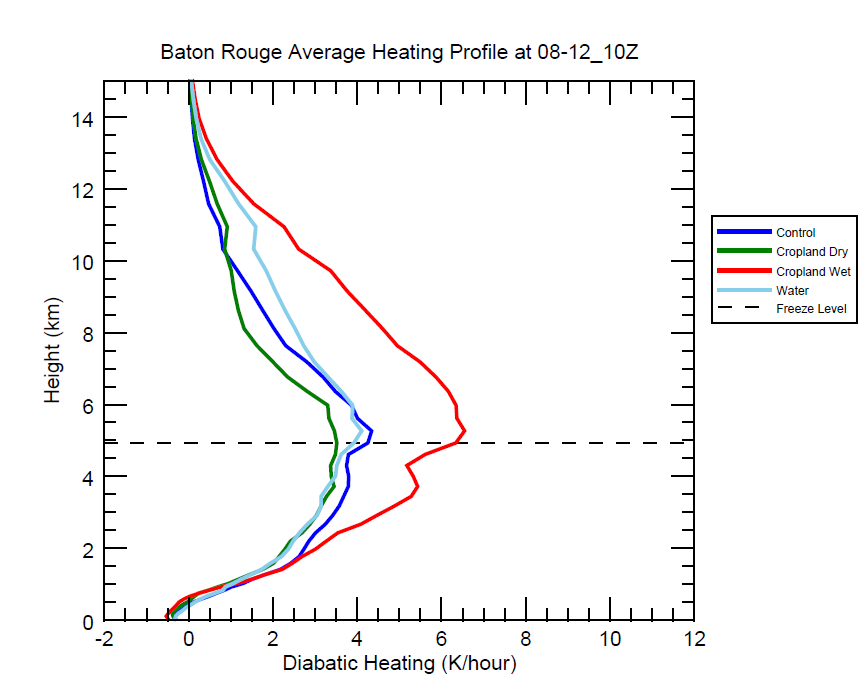


**Figure S5**. Area-averaged vertical profiles of latent heat release at 1000 UTC on August 12 for the different experiments.


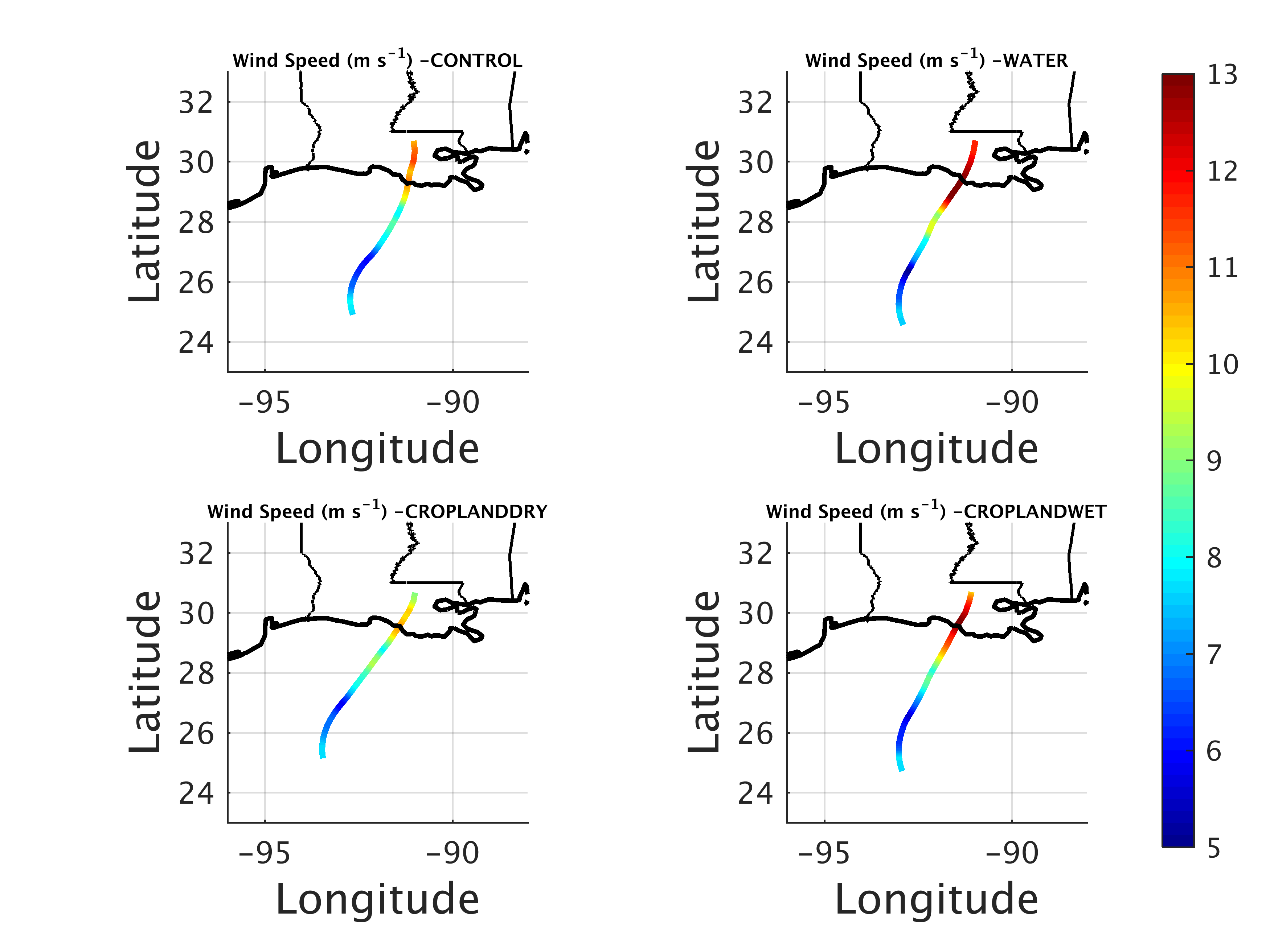


**Figure S6**. Mean values of wind speed along the mean position of 24-hour back trajectory intiated from points over Baton Rouge for control, open water, cropland dry, and cropland wet experiments. Maps were created using Matlab R2017a ( https://www.mathworks.com/products/new_products/release2017a.html).


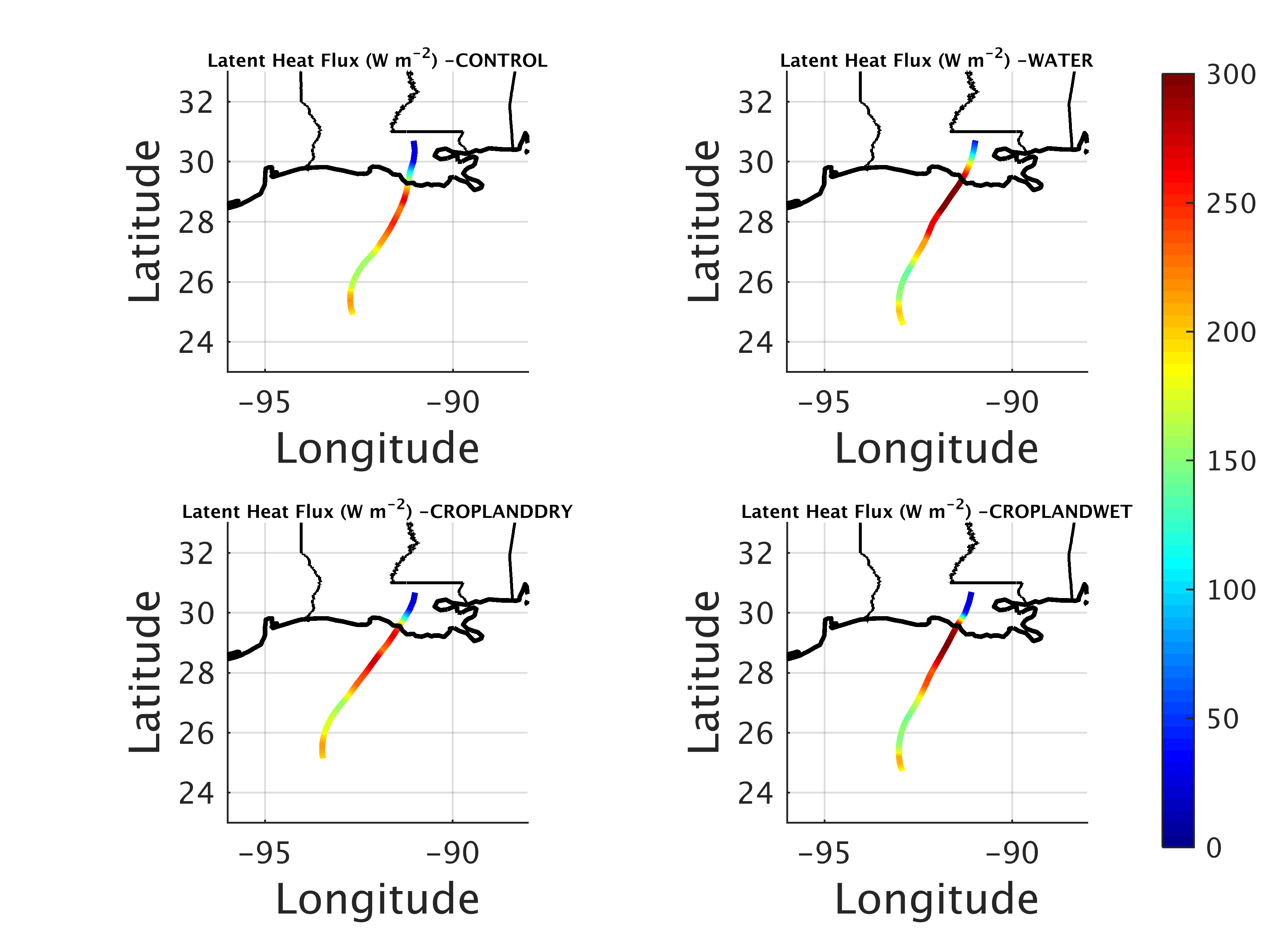


**Figure S7**. Mean values of latent heat fluxes along the mean position of 24-hour back trajectory initiated from points over Baton Rouge for control, open water, cronpland dry and cropland wet experiments. Maps were created using Matlab R2017a ( https://www.mathworks.com/products/new_products/release2017a.html).


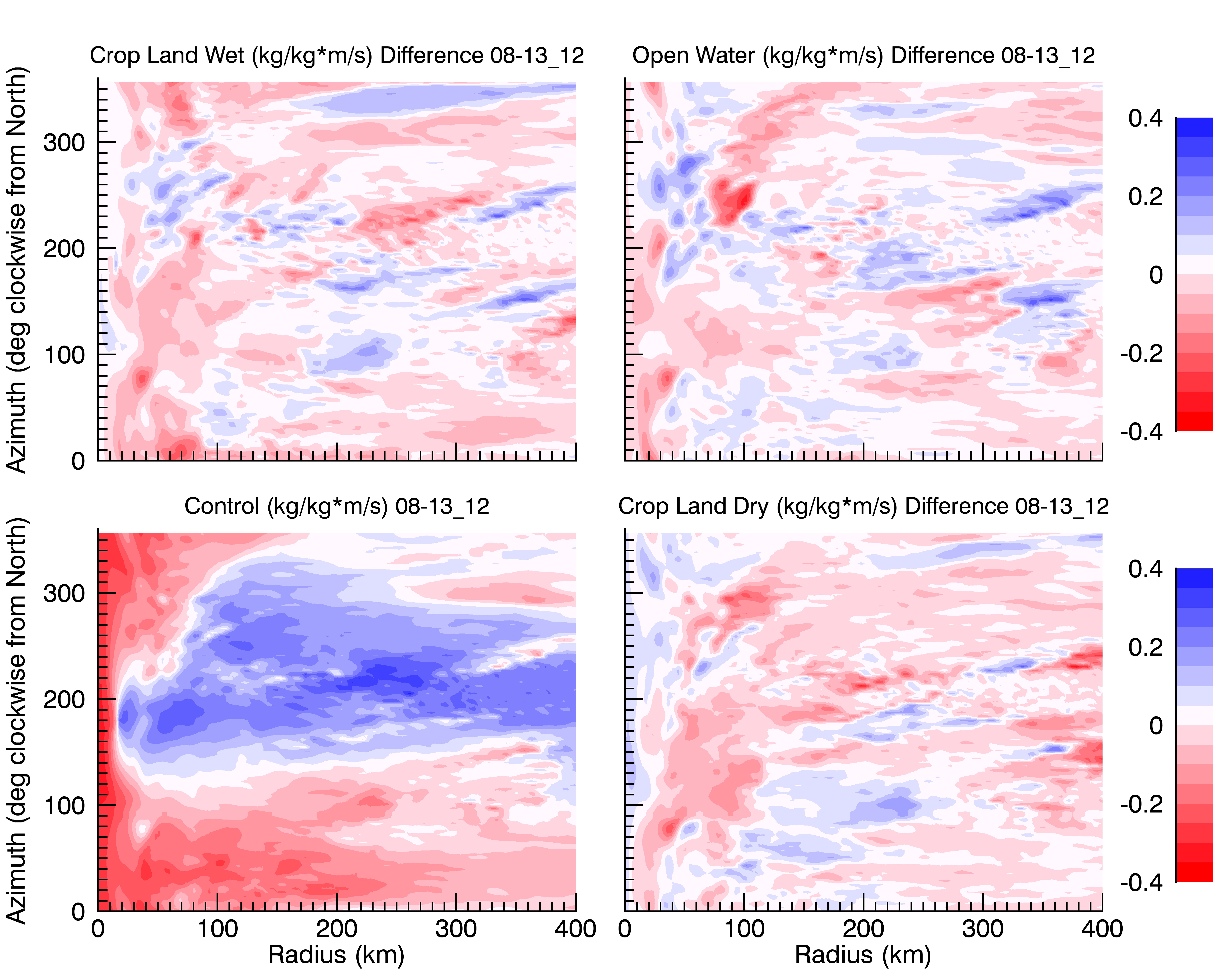


**Figure S8**. Radius-azimuth plot of moisture transport for the control simulation at 12 UTC, 13 August 2017 is shown on the bottom left paanel. Differencs in moisture transport between control and the cropland wet, open water and, cropland dry experiments are shown in top left, top-right and bottom left panles respectively.


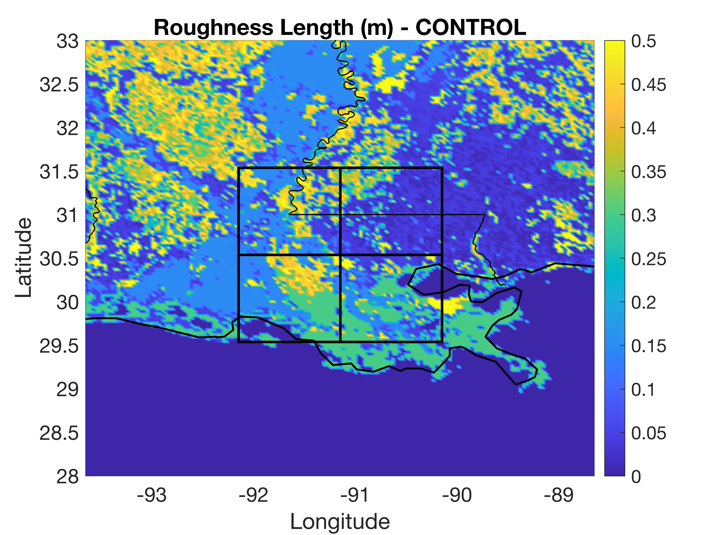

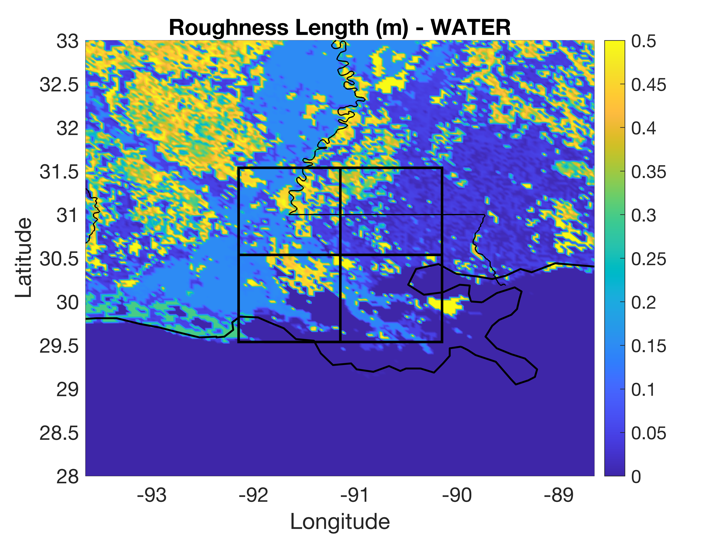

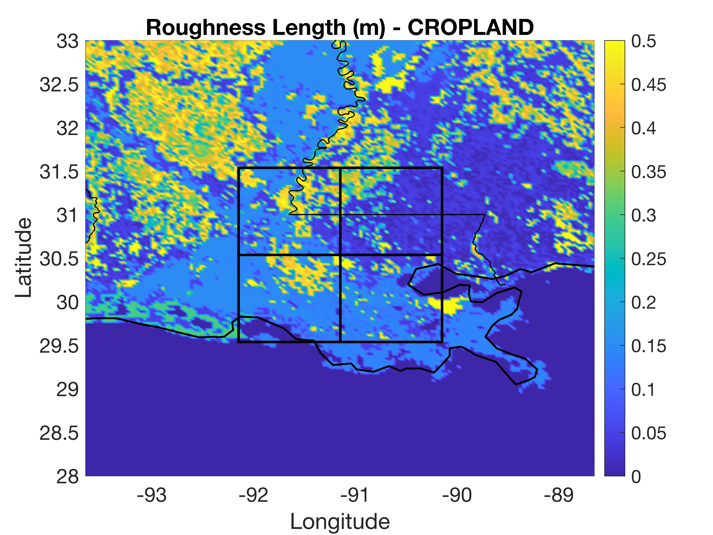


| 0.21 | 0.10 |
| --- | --- |
| 0.23 | 0.20 |

| 0.20 | 0.10 |
| --- | --- |
| 0.13 | 0.08 |

| 0.20 | 0.10 |
| --- | --- |
| 0.17 | 0.13 |

Figure S9. Spatial distribution of surface roughness used in the control, open water and, cropland (wet/dry) experiments are shown in left, middle and right panels respectively. The four quadrants used for analysis described in the text are outlined in black. The tables in the inset of each panel shows the average roughness length in each of the quadrant. Maps were created using Matlab R2017a ( <https://www.mathworks.com/products/new_products/release2017a.html>).


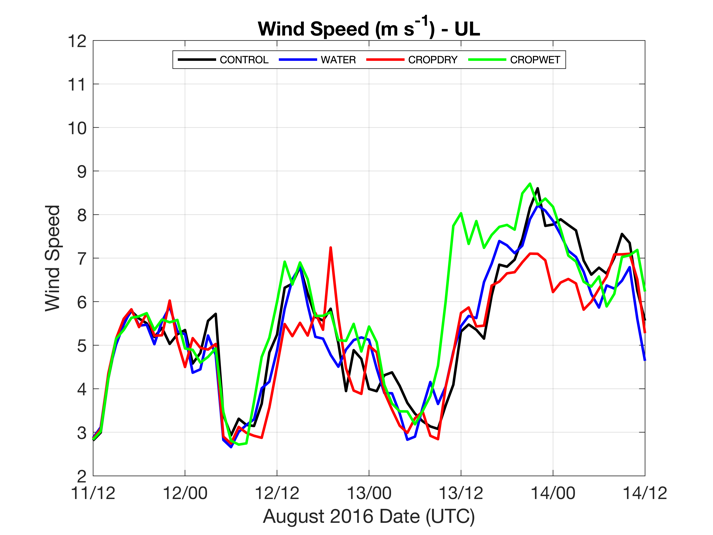

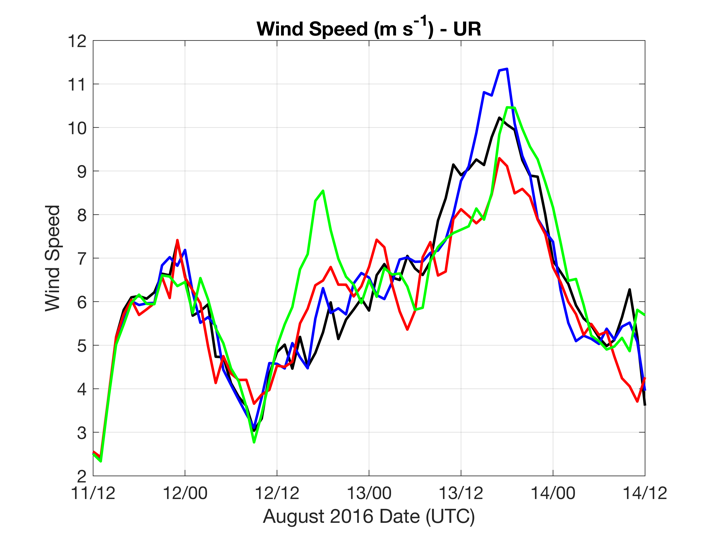

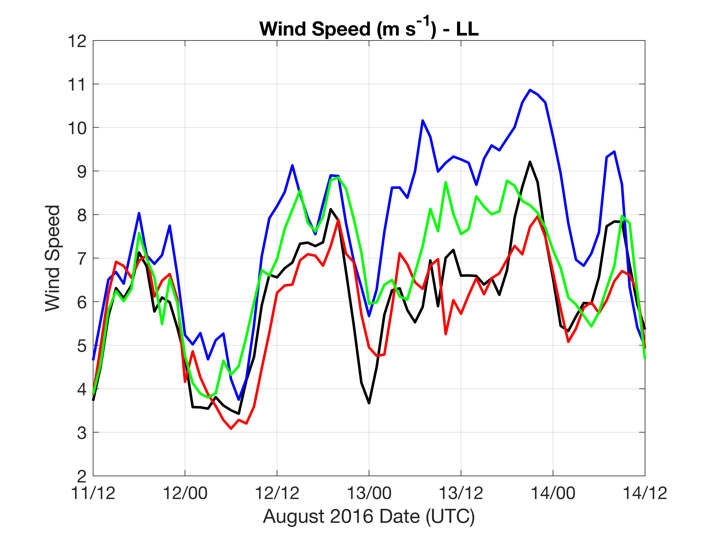

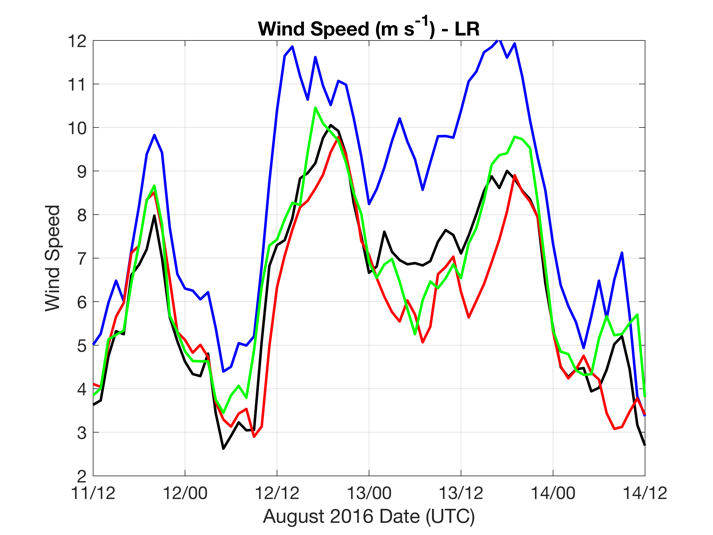


Figure S10. Time evolution of average surface wind speeds in each of the analysis quadrants shown in Figure S9.


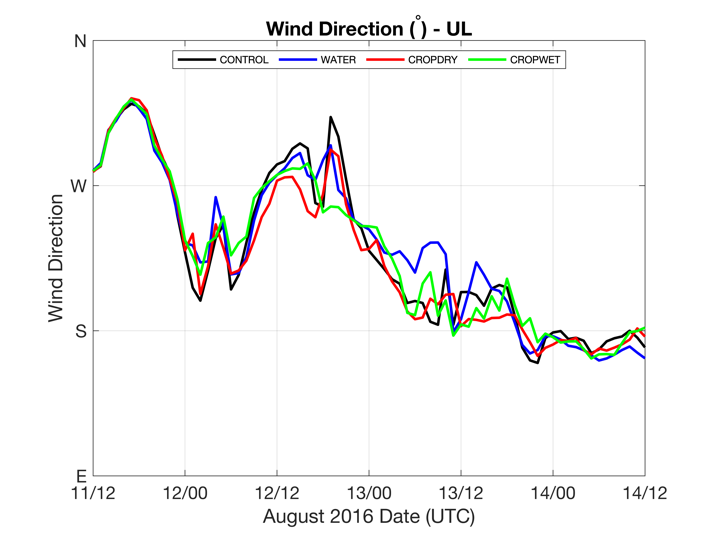

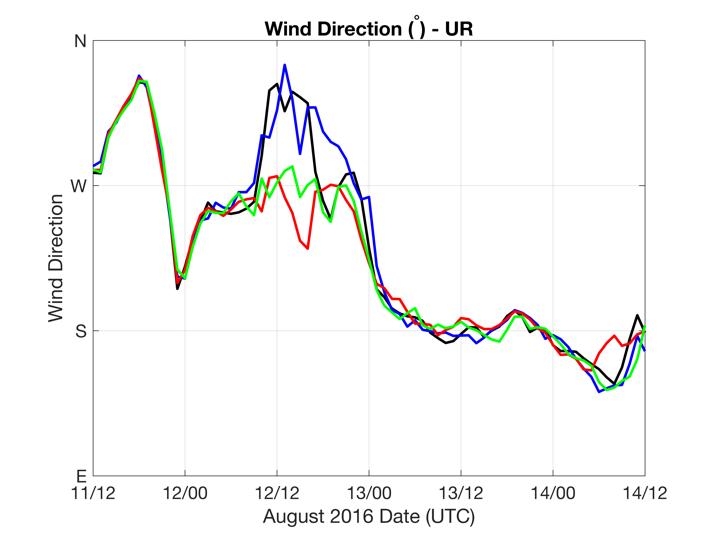

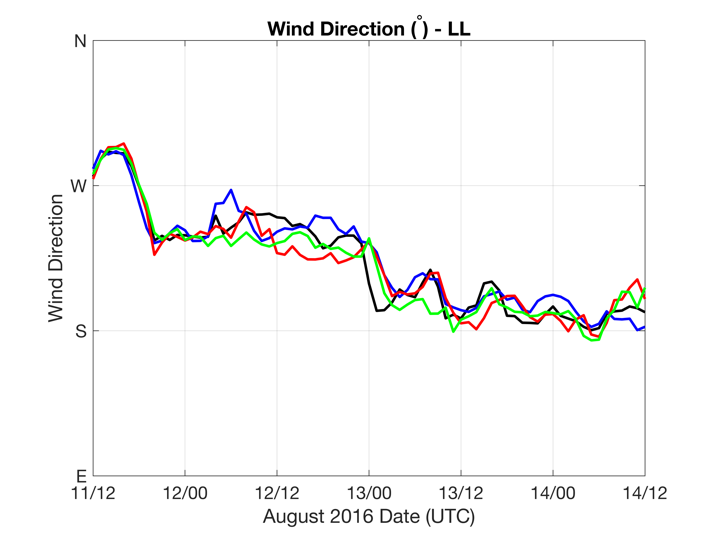

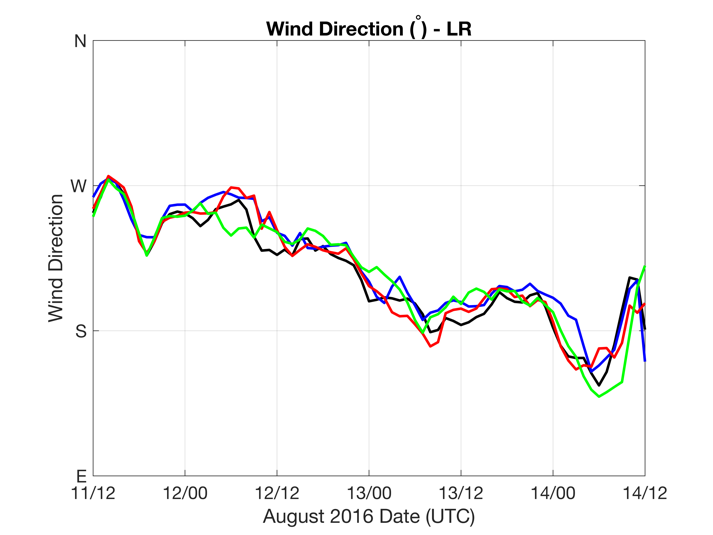


Figure S11. Time evolution of average surface wind direction in each of the analysis quadrants shown in Figure S9.


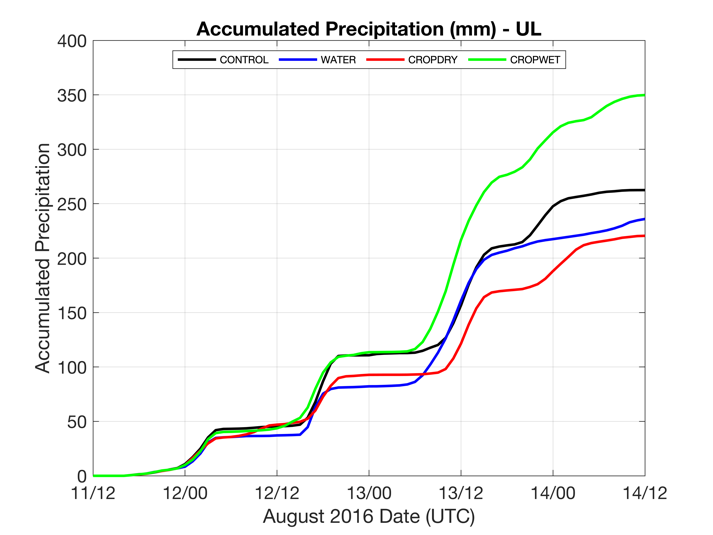

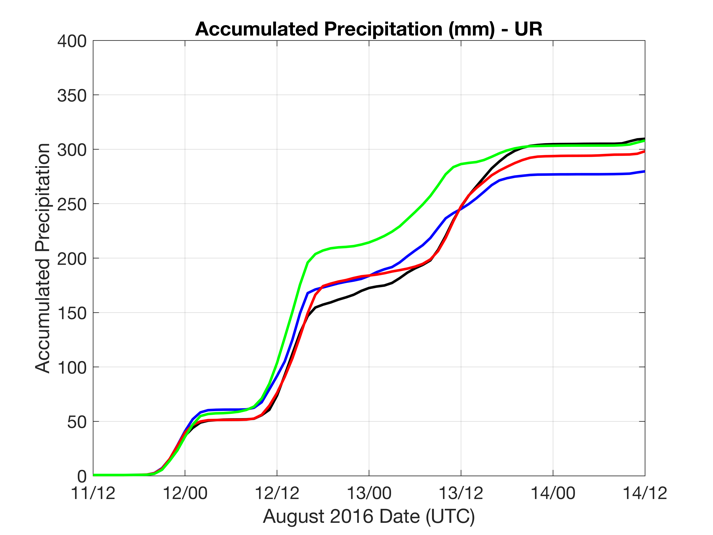

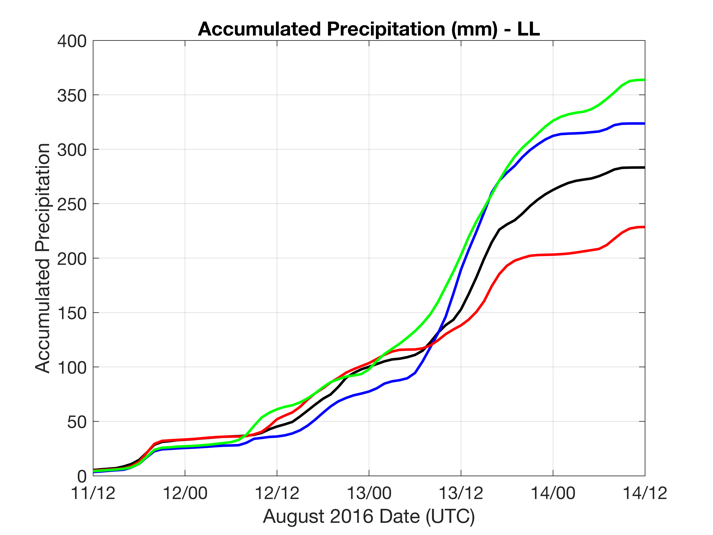

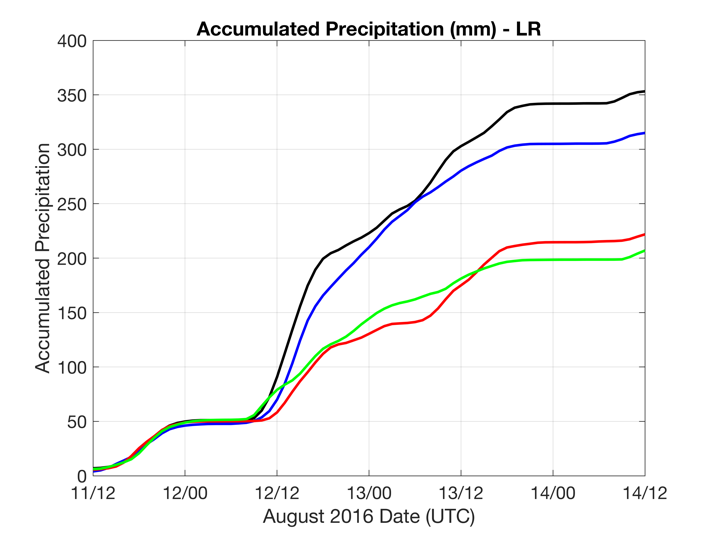


Figure S11. Time evolution of average surface rainfall accumulation in each of the analysis quadrants shown in Figure S9.
